# Cloud Enterprise Dynamic Risk Assessment (CEDRA): a dynamic risk assessment using dynamic Bayesian networks for cloud environment

**DOI:** 10.1186/s13677-023-00454-2

**Published:** 2023-05-17

**Authors:** Dawood Behbehani, Nikos Komninos, Khalid Al-Begain, Muttukrishnan Rajarajan

**Affiliations:** 1 School of Mathematics, Computer Sciences and Engineering, City, University of London, London, UK; 2 Kuwait College of Science and Technology, Kuwait City, Kuwait

**Keywords:** Cloud risk assessment, Quantitative risk-analysis, Dynamic Bayesian Network

## Abstract

Cloud computing adoption has been increasing rapidly amid COVID-19 as organisations accelerate the implementation of their digital strategies. Most models adopt traditional dynamic risk assessment, which does not adequately quantify or monetise risks to enable business-appropriate decision-making. In view of this challenge, a new model is proposed in this paper for assignment of monetary losses terms to the consequences nodes, thereby enabling experts to understand better the financial risks of any consequence. The proposed model is named Cloud Enterprise Dynamic Risk Assessment (CEDRA) model that uses CVSS, threat intelligence feeds and information about exploitation availability in the wild using dynamic Bayesian networks to predict vulnerability exploitations and financial losses. A case study of a scenario based on the Capital One breach attack was conducted to demonstrate experimentally the applicability of the model proposed in this paper. The methods presented in this study has improved vulnerability and financial losses prediction.

## Introduction

### Cloud services

Cloud computing adoption has been increasing rapidly among organisations of all sizes [1]. Amid COVID-19, enterprises have accelerated their digital transformation; thus, cybersecurity has become even a bigger challenge. Consequently, technology has become more essential in both our working and our personal lives. Companies have realised the importance of adapting to the market’s needs. As such, the adoption of cloud services and agile methodology has enabled the rapid delivery of services online. The mechanism of cloud services introduces many benefits for organisations, such as ease of deployment, on-demand scalability, wide accessibility and ease of management [2]. This has propelled the growth of cloud-based adoption and application. As adoption widens, so does the threat landscape. To cater to the dynamically changing landscape of threats towards the use of cloud services, effective security countermeasures should be implemented, selected based on risk and threat assessment [3].

### Risks in cloud services

**Fig. 1:**
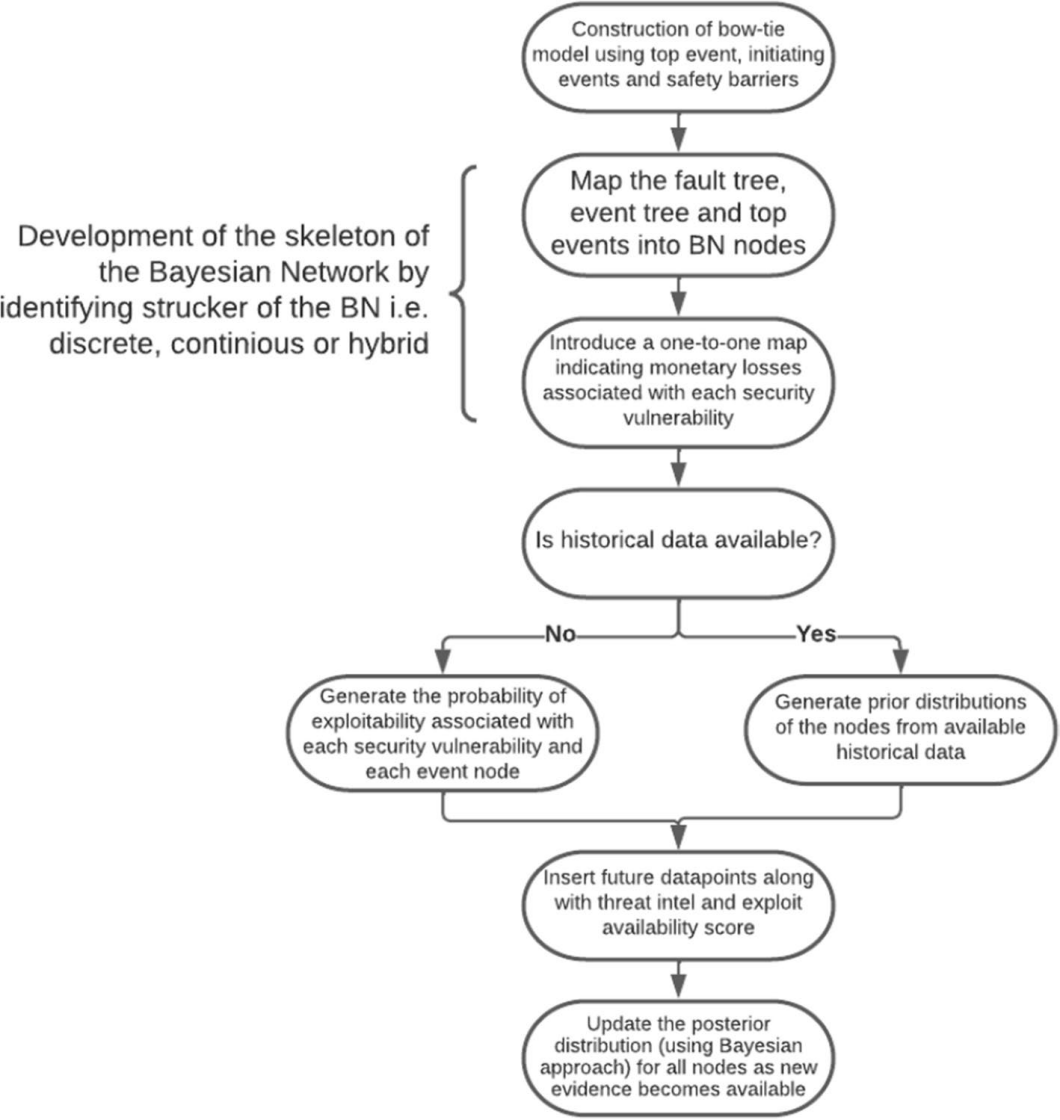
Schematic of the framework developed

Risks levels in cloud environments are prone fluctuation due to time-varying factors, such as the emergence of new vulnerabilities in safety barriers, installation of new software/components and misconfigurations. It is essential to quantify these time-dependent factors and their relations via robust calculation techniques. The outcome is then used to derive quantified estimates of risk that are mapped against a pre-defined risk criterion. Traditional risk assessment methods, such as quantitative risk assessment (QRA), and such methods as FT, ET and What-if analysis are limited in terms of incorporating new information or evidence, as these models cannot handle data scarcity and uncertainties. Therefore, it is important to focus research on the field of dynamic risk assessment, where risk is consistently emerging [3]. Dynamic Bayesian Network (DBN), an extended version of standard Bayesian Network (BN) with the concept of time, is a Probabilistic Graphical Model (PGM) that are potentially used to develop models from data or expert opinion using Bayes’ theorem. The model is ideal for a extensive range of functions such as prediction, deviation detection, diagnostics, reasoning, time series prediction and decision making under uncertainty. BN corresponds to a set of random variables, and their relationships are signified using a directed acyclic graph (DAG). The arcs signify the causal relationship between nodes, and the nodes represent the variables. The main node is called a ‘root node’. If there is an arc connected to another node, that is called a ‘parent node’, and if a the ‘parent node’ is connected to another node, that is called a ‘child node’, as seen in Fig. 1. All nodes in the BN are allotted an initial probability. The purpose of BN is to use the node probabilities and their relational dependencies to assess and use the provided evidence and posterior to update the distribution probabilities of the random variables. The calculation of the probabilities of the ‘child node’ is a blend of ‘parent node’ probability and conditional probability tables (CPT). This is considered the main protocol of the well-known ‘Bayes theorem’ of conditional probabilities [4]. The primary motivation of this paper is to address the issues described above. Thus, it presents a new model that quantifies the criticality of consequences and predicts risks in monetary terms. The model is updated as new information is acquired. Previous models suffer from a lack of historical data and have an inability to adapt in dynamic environments, so that the resulting risk assessments are subjective. To validate the proposed model, it was run against a scenario based on the Capital One breach in 2019. A dataset was collated with data from various sources. The corresponding bow-tie diagram was constructed using the components of the case study. Then a BN was developed to produce a dynamic risk assessment. It assumes no prior information about the probabilities. The results reveal that applying a combination of a bow-tie analysis based on a dynamic Bayesian network using threat intelligence and information about the availability of exploits in the wild can improve predictions of vulnerabilities and financial losses.

### Contributions

In the literature, most applied Bayesian network model in risk assessment studies are focused on areas such as aiming to predict drilling-related risks, asset failure in thermal power plant and industrial control systems, however do not take into consideration the monetary losses. To overcome this problem, this study aims to develop a cloud risk assessment framed that enables the assignment of monetary losses terms to the consequences nodes, thereby enabling experts to understand better the financial risks of any consequence. In this work, BT analysis has been converted into a dynamic BN network that estimates monetary losses for cloud environments. It is the first time such an analysis has been applied to cyber threats in cloud environments (previous usage of this method was in response to cyber attacks on physical systems, tank storage, etc). To ensure the feasibility of our study, a cloud service is simulated, and one undesired event is simulated based on the capital one breach case constructed. Our risk assessment model is developed and assessed against BN. The rest of this paper is organised as follows. Related work section demonstrates related work and justifies the reasons for using DBN. Our proposed risk assessment model is demonstrated in Framework for dynamic risk assessment section. The case study is then highlighted in Case study section to present our model. Finally, a discussion of the proposed model is presented in Discussion and results section and our work is concluded in Conclusion section.

## Related work

### Threat intelligence

Today’s cybersecurity threats have emerged, and traditional approaches based on heuristics and signatures are not very effective against dynamically changing threats known to be evasive, persistent and complex. Organisations must gather the latest cyber threat information to deter attacks in a timely manner [5]. Threat intelligence (TI) represents an actionable defence that aims to reduce the gap between the attack and the organisation’s defensive action [6]. TI can take on multiple forms and means. Numerous methods and tools offer TI feeds, such as IBM X-Force Exchange, CrowdStrike Intelligence Exchange and AlienVault OTX Pulse. In this study, we utilized the AlienVault OTX Pulse to retrieve pulse information regarding the vulnerabilities in scope of this study. This enables us to understand which vulnerabilities attackers are primarily pursuing.

### Risk assessment theory

The objective of risk assessment is to explore and determine threats and vulnerabilities in a particular area or scope to apply adequate mitigation controls that would minimise the risk to an appropriate level. The process is continuous to measure risk factors as they change and develop over a significant time [7]. The numerous impacts of risks on organisations adopting cloud services are either tangible or intangible losses, such as downtime, data loss and reputation jeopardy. However, in this study, we limit these impacts to system asset loss.

### Dynamic risk assessment

There are numerous cyber risk assessment frameworks aimed at exercising risk evaluation that were developed by governmental agencies, the cyber defence industry, and academic institutions. Nevertheless, such frameworks lack the mechanism to handle dynamically changing environment and cannot adapt their countermeasures and priorities to changes happening within inter-systems and external environments [8]. Another major issue in risk management involves real-time data scarcity and uncertainties to enable adequate risk calculation [9]. Data mining to predict the outcome of a cyberattack is a challenge especially in a situation where a series of factors may be involved in launching a malicious attack to cause losses. This requires a synthetic model that entails attack knowledge and system knowledge for analysing attack vectors and cybersecurity risks [10]. For example, [11] proposed the Bayesian attack graph method in risk assessment for predicting potential attacks. Such a model, including other models such as fault tree analysis (FTA), event tree analysis (ETA), bow-tie analysis (BTA), Markov chain analysis (MCA) and Bayesian network (BN) has a limitation as they require a deep insight into prior knowledge about attacks [9] or referred to as epistemic, a form of a uncertainty [12]. Another uncertainty can be referred to as aleatory uncertainty, where this is considered a nondeterministic nature of the events [13]. Therefore, we argue that a DRA addresses this issue by reassessing the risks by continuously updating the probabilities of events, as new informaton is fed and made available.

For example, [14] have proposed a dynamic risk assessment based on the 2.0 semantic version of STIX^TM^ for cyber threat intelligence that enables fetching indicators of compromise such as malicious URL, domain, and IP addresses. A dynamic risk assessment can be further considered a technique that takes consideration the effect of nonlinear interactions within inter-processes in its operational risk estimation. Thus, this type of technique can demonstrate a realistic prediction of the risks identified in sophisticated processes [15]. Another example of leveraging DRA is by [16], whereby they developed a conceptual dynamic quantitative risk assessment model for identifying and classifying accidents and the risk influence factors of barrier elements, which are physical objects that protect a target against a hazard. The limitation of their work is that they leveraged expert opinion in their methodologies which tend to be incomplete and subjective, thus the assessment results may be inaccurate. They may also potentially be incapable for predicting unknown attacks.

To address this issue, various scholars have proposed models that can compensate for the necessity of acquiring historical data. For example, the fuzzy probability Bayesian network approach replaces limited historical data with fuzzy probabilities and a fuzzy approximate dynamic inference algorithm [10]. Another example, is an automated intrusion response system that utilises dynamic risk assessment using fuzzy logic. This is merely because fuzzy logic lessens the level of uncertainty of risk factors [17]. (Table 1) A tabular depicts an overview of the literary study.

**Table 1:** Overview of the literary study related to DRA

Research Study	Modelling Approaches	Identified Gaps
[8]	System dynamics	Unable to handle dynamically changing environment
[10]	Fuzzy Probability Bayesian Network	Lack of attack knowledge and system knowledge for analysing attack vectors and cybersecurity risks
[11, 12, 13]	BN, life cycle assessment (LCA) and quantitative risk assessment (QRA), Delphi risk communication platform	Require a deep insight into prior knowledge about attacks to make the proposed model effective
[16]	Fuzzy DEMATEL-BN	Leveraging expert opinion in their methodologies which tend to be incomplete and subjective
[17]	Fuzzy logic	Lessens the level of uncertainty of risk factors

### Dynamic Bayesian networks

Bayesian Networks (BN) provide a useful mechanism in the risk analysis field due to their ability to model probabilistic data. Andrade et al. [18] states that dynamic BNs take into consideration temporal dependencies based on time. Basic BNs do not consider alterations in time or manage time-evolving environments; thus, dynamic BNs (DBNs) are ideal for handling time-dependent risk assessments. Li et al. [19] demonstrate how BNs have gained popularity because of their capabilities in predictive and diagnostic analyses. However, we are of the opinion that traditional BNs can only demonstrate relationships between variables at a specific time points, or for a specific period of time. They do not indicate temporal relationships between different times. To address this issue, DBNs can be used to present changes over time and relationships between a device’s current, past or future states. A DBN is an extension of a BN that introduces appropriate temporal dependencies to model the dynamic behaviour of attributes. Numerous inference algorithms are available for DBN modelling. In [20], the forwards-backwards inference and mutual information were used to model the Bayesian inference. In [20] and [21], the authors claim this is a major barrier to acquiring the precise probability of basic events related to a system target in a situation when objective data is scarce to predict probabilities pertaining to a target system. In addition, the availability of large data samples is a necessity of deep learning. Therefore, expert judgement is deemed an appropriate approach to obtain the occurrence probabilities of events. Although judgements obtained from expert opinions are subjective and susceptible to a margin of error, they are the ideal way forward. A DBN can also be leveraged using a FT, ET and BT, as these cannot handle the dynamic nature of operational risks. For example, [21] proposed a dynamic risk assessment methodology based on FT methods that is mapped into DBNs, however the domino effect, a phenomenon that is also referred to as a chain of accidents, which should be taken into consideration in risk assessments whereby primary events trigger a secondary or higher event is overlooked in this study. In [4] claims that a DBN requires a high number of simulation runs, merely because dynamic probabilistic risk assessment models require heavy computational power and system memory for running a vast number of simulations. Therefore, they proposed the use of a DBN with clustering analysis to enable a reduced number of simulation runs and the quantification of emerging system risk in a probabilistic manner for thermal-hydraulic simulation data. The approach was demonstrated with the mean shift clustering algorithm along with the bandwidth selection method. In addition, [22] asserts that the DBN has a strong advantage when handling uncertainty, because it is a directed acyclic graph (DAG) that describes the conditional probability relationship between parameters using probabilistic inference theory. However, the limitation in their methodology is that they are dependent on expert knowledge, thus are subjective and susceptible to a margin of error or may be rendered ineffective.

In this paper, a dynamic Bayesian network is leveraged in a case study that enables the prediction of vulnerability exploitation and financial losses. Our proposed model also has the capability to be updated as new information is attained and aids in quantifying the criticality of the consequences and predicting risks in monetary terms. We believe our work has demonstrated the importance of dynamic quantitative risk assessment for multiple reasons, including the absence of historical data, the inability to adapt to a dynamically changing environment and the need to avoid subjective risk assessment results as much as possible based on the gaps identified in the literature.

## Framework for dynamic risk assessment

The detailed process of the proposed methodology, Cloud Enterprise Dynamic Risk Assessment (CEDRA), is shown in Fig. 1.

1. **Construction of a bow-tie (BT) model (top event, initiating events and safety barriers)** BT has been widely employed as a graphical approach to represent an end-to-end accident scenario, from its causes to its consequences. The top event is placed in the centre, and on the left-hand side is a ET that identifies the potential events causing the critical event. On the right-hand side is an event tree that depicts the possible consequences of the critical events based on the safety barrier’s success or failure. To simplify this, an example of a bow-tie top risk sources and consequences is as following:
  Fireplace (top threats) and death (top consequence).
  Electrical fault (top risk source) and equipment damage (top consequence).
  ... etc.
2. **Developing of the skeleton of the Bayesian network from BT** Starting out with a BT, a Bayesian network (BN) needs to be constructed. A BN is a DAG that is broadly used in risk and safety analyses, inspired by probability and uncertainty. Graphically, a BN’s structure is created based on a FT and an ET in such a manner that the top event and the causes shown in the FT are demonstrated by nodes, while its relationship is depicted through arcs in the BN. OR gates and AND gates are used to present relationships between the causes and the top event in the ET. The process of mapping a ET into a BN comprises nodal representations of the barriers. Each node generally has discrete outputs of nodes. For equipment that operates continuously, nodes can take continuous values. Therefore, matters such as the probability of failure within a specified time period and the time to failure (TTF) for equipment under continuous operation are taken into consideration. The failure rate is considered constant or time dependent. When the nodes have discrete outputs, we use a discrete node BN with marginal prior probabilities, and when the nodes have continuous outputs, we use a marginal distribution for the nodes (Weibull, exponential or gamma distributions). For example, each top threat and consequence is placed within a node. An arc signifying causal relationship is drawn between them.
3. **Incorporating monetary loss terms in the BN** This study discusses a method for incorporating monetary losses resulting from a failure (or the triggering of a top event). Each security vulnerability is associated with a monetary loss. The total financial loss associated with the breach of an event would be given by the probability of a breach of an event node multiplied by the financial loss associated with that node. The financial loss associated with a node can be calculated from the financial loss associated with each security vulnerability. The financial loss limited to asset purchasing cost has been calculated by the following formula:
  $$\begin{aligned} F(t)=\sum ^6_{j=0}{{P_{exploitability,d}(v}_j,t).\ a(v_j)} \end{aligned}$$
  where F(t) is the financial loss at time t. Pexploitability,d (v_j_,t) is the dynamic probability of exploitability (considering EA and TI scores) of vulnerability vj (where j varies frorm 0 to 6), and a(vj) is the maximum asset loss of vulnerability vj. For simplicity, we divided the monetary losses based on asset value. We can expect higher risk consequences leading to higher monetary class nodes and vice-versa over time. Please refer to Table 2
4. **Construction of the complete BN** Once the initiating events, monetary loss nodes and top event and consequences are identified and the skeleton of the BN is determined, the final Bayesian network can be developed. When expert opinion is not available, step 3 can be skipped, and a BN is constructed without considering the risk influence factors.
5. **Check whether historical data are available** Imagine a BN with n variables A1, A2,..., An. The joint probability distribution of the BN is merely presented as
  $$\begin{aligned} P\left( A_1,A_2,\ \dots .,\ A_n\right) =\ \prod ^n_{i=1}{P\left( A_i\left| Parents\left( A_i\right) \right. \right) } \end{aligned}$$
  [23] where Parents(Ai) denote the set of parent nodes of the node Ai. If A and B are two random events with a prior probability P(A) and P(B), the posterior probability of event A occurring, given that B has occurred, can be determined by Bayes’ rule
  $$\begin{aligned} P\left( A|B\right) =\frac{P\left( A\right) P\left( B|A\right) }{P(B)} \end{aligned}$$
  [23] If historical data are not available, we construct the initial CPT using the CVSS scores of all the security vulnerabilities used in constructing the event graph. We will use the static scores (access vector, attack vector, permission for intervention and user interaction) to create a static probability of exploitability values for the initial CPT. As more evidence is added, including dynamic data, such as threat intelligence scores and exploit availability information scores, the CPTs will be updated accordingly.
6. **Evaluation of the dynamic risk profile** When no historical data are present, the BN has no information on how to connect initiating events with consequences. As data are added, the CPT can be updated. The CPT is updated using the following:
  Dynamic CVSS scores (threat intelligence scores, exploit availability scores)
  - A Bayesian model that takes as input historic CPT values and new evidence (for example, whether a security node was breached or a top event was triggered) and uses that to update the CPT tables

**Table 2:** DRAs modelling based on Formulas and Framework

Asset	Value (USD)
Web Server	0
SQL Server	0
Gateway Server	10,000
Application Server	10,000

### Risk assessment model

The vulnerabilities of the cloud environment are commonly identified using numerous scanning tools and the inputs of system experts (manual assessment), including historical data on previous incidents by attackers. System vulnerabilities can also be identified on the basis of asset information from the Common Vulnerabilities and Exposures (CVE) database of asset/product information such as product names and versions. The third version of the Common Vulnerability Scoring System (CVSS) developed by the National Institute of Standards and Technology (NIST) is aimed at defining the characteristics of vulnerabilities and generating a numerical score to reflect the severity of a vulnerability. The score consists of a combination of parameters, including access vector (AV), access complexity (AC), and authentication (AU). Further information can be obtained from the CVSS v3.1 user guide [reference]. Zangeneh and Shajari [24] proposed to assess the probability of vulnerability exploitation using the equation

$$\begin{aligned} P_{exploitability,s}=\frac{C}{F}\times AV\times AC\times PR\times UI \end{aligned}$$

In this paper, we adopt this method while introducing two new parameters: exploit availability (EA) and threat intelligence (TI).Therefore, the successful exploitation of a specific vulnerability after the introduction of EA and TI is as follows:

$$\begin{aligned} P_{exploitability,d}=\frac{C_0}{F_0}\times AV\times AC\times PR\times UI\times TI\times EA \end{aligned}$$

where C and C_0_ denote the exploitation factor and F and F_0_ the upper limit of the exploitation score. As for AV, AC, PR and UI, they demonstrate the static metrics: AV stands for access vector, AV is the metric of the attack vector, PR is the metric of the required permissions for intervention and the metric UI is the user interaction, respectively. The dynamic metrics are represented by TI and EA-the threat intel and exploit availability scores, respectively. EA was set at a value of 0.33 when available and 0.66 when not available. As for TI calculation, values < 10 were set at 0.45; between 10 and 45, at 0.50; and >45, at 0.55. To construct the initial BN at t = 0, we assume no knowledge of the dynamic BN and hence use P_exploitability,s_ to generate the initial CPT values. In our Bayesian network, certain event nodes can be caused only by the occurrence of all security event nodes; in this case, we use the AND gate to describe this relationship that has been adopted from [25].

$$\begin{aligned} P\left( X_i | Parent\left( X_i\right) \right) =\ \left\{ \begin{array}{c} 0,\ \ if\ \exists X_j\in Parent\left( X_i\right) ,\ X^s_j=0\ \\ P\left( \ \bigcap _{X^s_j=1}{e_i}\right) =\ \prod _{X^s_j=1\ }{P\left( e_i\right) ,\ \ otherwise}\ \end{array} \right. \end{aligned}$$

When the occurrence of any security event out of the many possible events triggers a warning, we use the OR gates to describe the relationship (for example, the relationship between v0 and v6 going to the node ‘block at firewall’ is described by OR logic).

$$\begin{aligned} P\left( X_i | Parent\left( X_i\right) \right) =\ \left\{ \begin{array}{c} 0,\ \ if\forall \ X_j\in Parent\left( X_i\right) ,\ X^s_j=0\ \\ P\left( \ \bigcup _{X^s_j=1}{e_i}\right) =\ 1-\prod _{X^s_j=1\ }{(1-P\left( e_i\right) ),\ \ otherwise}\ \end{array}\right. \end{aligned}$$

## Case study

The selected scenario is based on the Capital One breach that occurred in 2019. Capital One is the fifth largest consumer bank in the U.S. and is considered one of the early banks to adopt the cloud computing environment from Amazon, which played a key role in the 2019 incident. The bank’s objective was to reduce its on-premise data centre operation and expand its cloud service footprint. They also worked closely with AWS to craft a security model to achieve a robust, secure cloud operation. In 2019, the bank announced that adversaries gained unauthorised access and acquired some personal information from Capital One credit card customers [26]. CloudSploit published an incident analysis report indicating that the compromise of the vulnerable system was achieved by executing a Server-Side Request Forgery (SSRF) attack that exploited a misconfigured web application firewall (WAF) known as “ModSecurity”. In a typical SSRF attack, the attacker’s objective is to initiate a unauthorised connection to internal-only services within the organisation’s infrastructure in order to gain access to internal systems [27]. Based on our research, the vulnerability known as CVE-2019-2828 was the cause of the exploitation. The vulnerability was added into the National Vulnerability Database few days post the incident. The vulnerability has a base score of 9.6, implying that its requires minimal effort for exploitation that enables unauthenticated adversary with network access via non-secure HTTP to jeopardise the WAF component [28]. The dataset used in this study was generated using a python script that fetches vulnerabilities from NVD, exploit-db.com (to retrieve information regarding exploitability availability in the wild), and AlienVault OTX Pulse to retrieve threat intelligence pulse information regarding the vulnerabilities in the scope of the study. We ran the dataset generation tool for a month to retrieve sufficient data. The vulnerability related to the Capital One incident was added synthetically onto the dataset.

The corresponding bow-tie diagram was constructed using the components of the case study. Each of the vulnerabilities from v0 to v6 should be associated with an exploitability probability, where P_exploitability_ represents the probability of successful exploitation. We are constructing a BN with dynamic risk assessment that assumes no prior information on the probability values. As vulnerabilities occur, the probability values for the different events will be recorded. As these probability values are recorded, the conditional probability tables would be updated (how to construct CPT tables is shown in ([25], Tables 8–14)

Tables 2, 3, 4, 5 and 6 show the conditional probability distributions of the different event nodes (WS: web server, SS: SQL server, GS: gateway server, AS: admin server). These nodes were taken from the topology of the test network in [29]. The irrelevant nodes that were not used in our case study were removed.

**Table 3:** Probability that one vulnerability is successfully exploited

ID	CVE ID	AV	AC	AU/PR	UI	TI	EA	P_exploitability_
v0	CVE-2019-2828	N(0.85)	L(0.77)	N(0.85)	R(0.62)	0(0.45)	No(0.5)	0.596
v1	CVE-2021-32791	N(0.85)	H(0.44)	N(0.85)	N(0.85)	0(0.45)	No(0.5)	0.467
v2	CVE-2021-1636	N(0.85)	L(0.77)	L(0.62)	N(0.85)	1(0.45)	No(0.45)	0.596
v3	CVE-2021-38639	L(0.55)	L(0.77)	L(0.62)	N(0.85)	-0.45	-0.45	0.351
v4	CVE-2021-36965	N(0.85)	L(0.77)	N(0.85)	N(0.85)	-0.45	-0.45	0.744
v5	CVE-2020-0670	L(0.55)	L(0.77)	L(0.62)	N(0.85)	0 (0.45)	No (0.45)	0.386
v6	CVE-2020-0720	A(0.62)	L(0.77)	H(0.5)	N(0.85)	0 (0.45)	No (0.45)	0.386

**Table 4:** Conditional probability distribution of the web server (WS) node

WS	WS_0_	WS_1_
P(WS=WSs | v00, v10 )	1	0
P(WS=WSs | v01, v10 )	0.429	0.571
P(WS=WSs | v00, v11 )	0.429	0.571
P(WS=WSs | v01, v11 )	0.184	0.816

**Table 5:** Conditional probability distribution of the SQL server (SS) node

SS	SS_0_	WS_1_
P(SS = SSs | v00, v10, v20 )	1	0
P(SS = SSs | v00, v10, v21 )	1	0
P(SS = SSs | v00, v11, v20 )	1	0
P(SS = SSs | v01, v10, v20 )	1	0
P(SS = SSs | v00, v11, v21 )	0.696	0.304
P(SS = SSs | v01, v10, v21 )	0.696	0.304
P(SS = SSs | v01, v11, v20 )	1	0
P(SS = SSs | v01, v11, v21 )	0.566	0.434

**Table 6:** Conditional probability distribution of the gateway server (GS) node

GS	GS_0_	GS_1_
P(GS = GSs | v00, v10, v30 , v40, v50)	1	0
P(GS = GSs | v01, v10, v30 , v40, v51)	0.73	0.27
P(GS = GSs | v01, v10, v31 , v40, v50)	0.73	0.27
P(GS = GSs | v01, v11, v30 , v40, v51)	0.615	0.385
P(GS = GSs | v00, v10, v31 , v40, v51)	1	0
P(GS = GSs | v01, v10, v31 , v40, v51)	0.588	0.412
P(GS = GSs | v01, v11, v31 , v41, v51)	0.184	0.816

Tables 4, 5 and 6 only show a subsample of the very many possibilities. There are 32 possibilities in Table 3, 64 possibilities in Table 4 and 128 possibilities in Table 5. Here, we have shown only a small subset related to different risk scenarios. Let us consider a scenario in which the evidence chain goes via v0, v2, v3 and v6. In this case, the probability of the web server being compromised is P(WS = WS1 | v01, v10 ) = 0.571.

The probability of the gateway server being compromised is P(GS = GS1 | v01, v10, v31, v40, v50) = 0.270. This value is obtained by P_exploitability_ (v0) * P_exploitability_ (v3) using the AND gate relationship.

The probability of the admin server being compromised is P(AS = AS1 | v01, v10, v31, v40, v50, v61 ) = 0.116. This value is calculated by P_exploitability_ (v0) * P_exploitability_ (v3) * P_exploitability_ (v6) (Table 7).

**Table 7:** Conditional probability distribution of the admin server (AS) node

AS	AS_0_	AS_1_
P(AS = ASs | v00, v10, v30 , v40, v50 , v60 )	1	0
P(AS = ASs | v01, v10, v31 , v40, v50 , v60 )	1	0
P(AS = ASs | v01, v10, v31 , v40, v50 , v61 )	0.884	0.116
P(AS = ASs | v01, v10, v30 , v40, v51 , v61 )	0.884	0.116
P(AS = ASs | v01, v11, v31 , v40, v51 , v61 )	0.748	0.252
P(AS = ASs | v01, v11, v31 , v41, v51 , v61 )	0.65	0.35

The probability for the SQL server being compromised is P(SS = SS1 | v01, v10, v21 ) = 0.303. This value is calculated by P_exploitability_ (v0) * P_exploitability_ (v2).

The probability of the top event (TE) node triggering is the combined probability of the admin server being compromised and the SQL server being compromised, which is P(TE = TE1 | v01, v10, v21, v31, v40, v50, v61 ) = P(AS = AS1 | v01, v10, v31, v40, v50, v61 ) * P(SS = SS1 | v01, v10, v21 ) = 0.116*0.416 = 0.035 (Table 8).

**Table 8:** Conditional probability distribution of node TE (Top event)

TE	TE_0_	TE_1_
P(TE = TEs | v00, v10, v20, v30 , v40, v50 , v60 )	1	0
P(TE = TEs | v01, v10, v21, v31 , v40, v50 , v61 )	0.965	0.035
P(TE = TEs | v01, v10, v21, v31 , v41, v50 , v61 )	0.926	0.074
P(TE = TEs | v00, v11, v21, v31 , v41, v50 , v61 )	0.926	0.074

### Dynamic updating of CPTs

Initially, when t = 0, we use P_exploitability,s_ to define CPTs, as shown above. With time, we collect a series of data points that provide information on threat intel scores and exploit availability. In addition, at each data point, we look for evidence of whether a security node was breached or a top event was triggered. This evidence is used to update the Bayesian probabilities of each security vulnerability and the subsequence event nodes. We have a series of 33 data points with dynamic information available. We will randomly insert evidence of WAF misconfiguration being exploited between these data points to see how the CPTs change over a given period and how the financial asset losses correspondingly change with time.

## Discussion and results

**Fig. 2:**
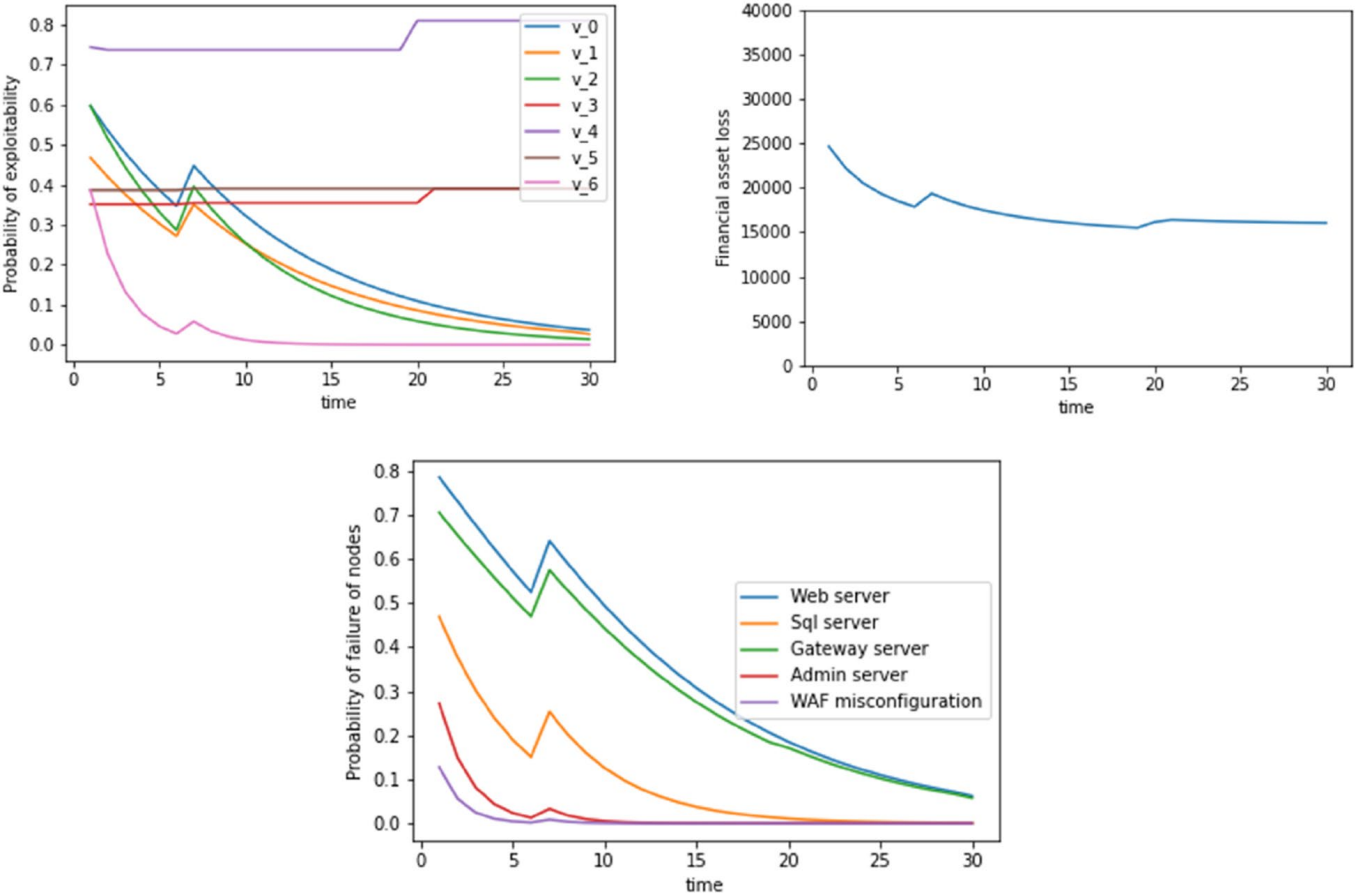
Probability of exploitability, financial asset loss, probability of failure of nodes with time where WAS misconfiguration is exploited as evidence at t=6. Dynamic update includes a Bayesian update plus the inclusion of TI and EA scores

This section provides an attack scenario based on the capital breach case to evaluate our model. We utilised 33 datasets where the WAF configuration (top event) was exploited as evidence at t=6. Therefore, we initially started with the probability of exploitability defined by P_exploitability,s_ in the above section. At each time step, the code reads the dataset sheet to look for the dynamic exploitability scores: TI and ES. It also looks for evidence as to whether the top event (WAF misconfiguration) was triggered or not. We synthetically added evidence at a user-defined timestep for the WAF misconfiguration to be exploited. In the three graphs of Fig. 2, this has been done at t=6 units.

**Fig. 3:**
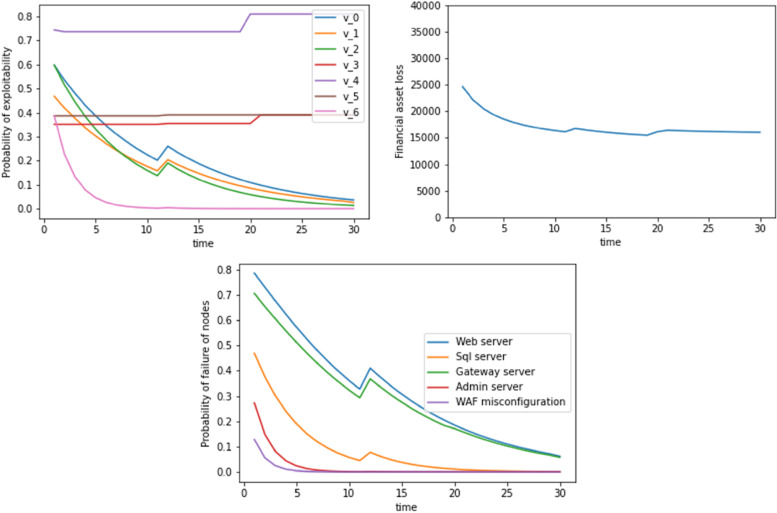
Probability of exploitability, financial asset loss, and probability of failure of nodes with time where WAF misconfiguration is exploited as evidence at t=11. The dynamic update includes a Bayesian update plus the inclusion of TI and EA scores

At time t<6 units, the code looks through the datasheet and finds no evidence of exploitation, and it hence updates the probability of exploitability of the security vulnerabilities (v0, v1, v2...). After t=6, there is an unexpected jump in the exploitability probability values, the corresponding asset losses, and the probabilities of failure of the different servers. For t>6, the same process is again repeated, where the code looks at the TI and ES scores and identifies whether the WAF misconfiguration was exploited. At t=25, we again see a jump in exploitability values. This again means that the TI and EA scores might have increased at those times. Figure 3 shows another example where WAF misconfiguration was exploited at t=11.

**Fig. 4:**
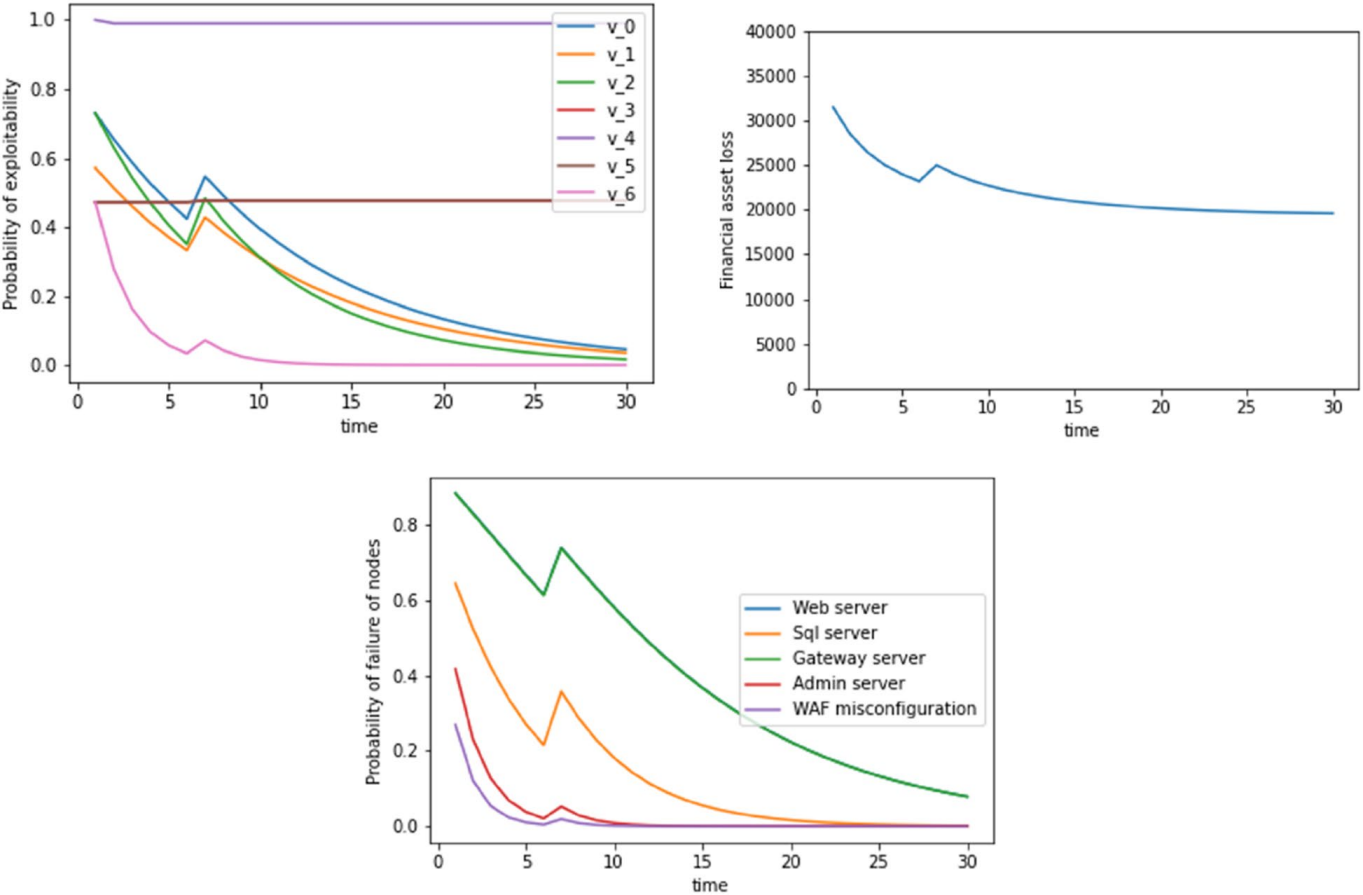
Probability of exploitability, financial asset loss, probability of failure of nodes with time where WAF misconfiguration is exploited as evidence at t=6. Dynamic update includes a Bayesian update without the TI and EA scores

**Fig. 5:**
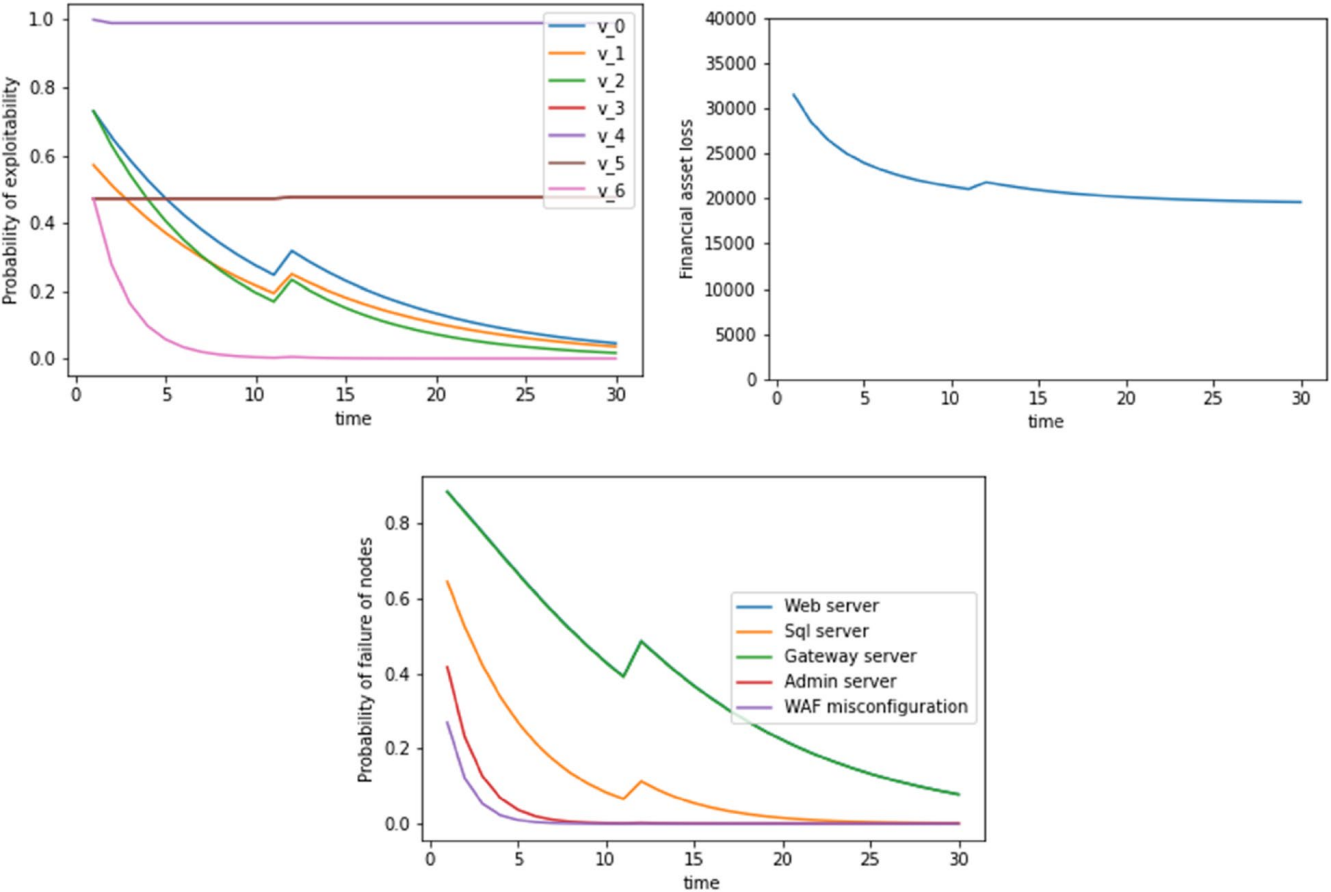
Probability of exploitability, financial asset loss, probability of failure of nodes with time where WAF misconfiguration is exploited as evidence at t=11. Dynamic update includes a Bayesian update without the TI and EA scores

In Figs. 4 and 5, we see the dynamic update occurring with a Bayesian approach without considering the TI and EA score update. In this case, all the 3 figures are relatively smooth except a peak that occurs at t=6 and t=11 time units respectively. This is because at these time instances, we find evidence for a WAF misconfiguration as has been shown in Figures 1 and 2. Due to the absence of TI and EA scores, we do not see the increase in the exploitation probability of exploitability financial asset loss, failure of nodes (at t=21) units.

Another example where WAF misconfiguration was exploited at t=11 is shown in Fig. 3.

The results clearly indicate that the model can incorporate new information and handle data scarcity and uncertainties, so that it is suitable for protecting cloud services. Cloud services are particularly susceptible to fluctuations due to time-varying factors, the occurrence of new vulnerabilities and misconfigurations. The Capital One breach occurred because of a WAF misconfiguration.

## Conclusion

Risk assessment is an integral part of risk management. It is aimed at proactively identifying threats and vulnerabilities that target assets and applying mitigation strategies to reduce the risks to an acceptable level. This paper proposes the Cloud Enterprise Dynamic Risk Assessment (CEDRA) model that uses CVSS, threat intelligence feeds and exploitation availability in the wild using dynamic Bayesian networks to predict vulnerability exploitations and financial losses. The probability of successful exploitation and financial losses is calculated by identifying CVE for each asset and then constructing a bow tie model based on the Capital One breach use case of 2019. The conditional probability distributions are achieved by AND and OR logic gates. The framework is based on a dynamic Bayesian network that facilitates an underlying process of continuously identifying and assessing risks in the cloud environment. The current study has shown that combination of bow-tie analysis, including dynamic Bayesian network, threat intelligence and and information about exploitation availability in the wild has improved vulnerability and financial losses prediction. However, the work could be further enhanced by introducing data asset value, as it is currently limited to asset purchasing cost and location of the asset.

## Data Availability

Not applicable.
